# Impact of Phacoemulsification Parameters on Central Retinal Thickness Change Following Cataract Surgery

**DOI:** 10.3390/diagnostics13172856

**Published:** 2023-09-04

**Authors:** Lars H. B. Mackenbrock, Isabella D. Baur, Grzegorz Łabuz, Gerd U. Auffarth, Ramin Khoramnia

**Affiliations:** Department of Ophthalmology, University Hospital Heidelberg, 69120 Heidelberg, Germany

**Keywords:** cataract, macula, retina, phacoemulsification, pseudophakic cystoid macula edema, retinal thickness, optical coherence tomography

## Abstract

Cataract surgery can lead to inflammatory processes in the retina due to its invasive nature, resulting in prolonged recovery times and reduced functional outcomes. The aim of the current study is to explore the impact that phacoemulsification parameters have on macular thickness following surgery. This prospective single-center study enrolled 46 healthy patients (46 eyes) who underwent uneventful cataract surgery. Retinal thickness was assessed using optical coherence tomography (OCT) preoperatively, as well as 1, 4, and 12 weeks after surgery. The macula was divided into a central (CMT), inner (IMT), and outer ring (OMT). Cataract density was automatically determined using an anterior segment OCT and a custom MATLAB script. Corrected distance visual acuity (CDVA), intraocular pressure (IOP) as well as cumulative dissipated energy (CDE), ultrasound time (UT), and fluids used during phacoemulsification were recorded. Retinal thickness and volume increased significantly following cataract surgery, reaching its maximum 4 weeks post-operatively. Statistically significant correlations were found between the CDE and IMT, OMT and retinal volume change (r_IMT_ = 0.356, r_OMT_ = 0.298, rvolume = 0.357 with *p* < 0.05) as well as between the ultrasound time and IMT, OMT, and retinal volume change (r_IMT_ = 0.369, r_OMT_ = 0.293 and r_volume_ = 0.409 with *p* < 0.05). Changes in CMT did not correlate with any surgical metrics. Additionally, no correlation was found to the amount of fluid used, whether CDVA or IOP. However, a link between nuclear cataract density and changes in OMT (r = 0.310, *p* < 0.05) was established. How ultrasound energy impacts the choroidea, and to what extent retinal metabolism changes after surgery, needs to be explored in future studies.

## 1. Introduction

Cataract surgery is among the most frequent surgical interventions worldwide. Today, it is generally considered to be safe and efficient, being able to restore excellent post-operative functional results [1]. Thanks to recent innovations in phacoemulsification technics, smaller incisions and shorter operation time, surgical strain on the eye has been reduced and post-operative recovery times have been shortened. Nevertheless, the intervention still leads to a secretion of inflammatory mediators. These diffuse from the anterior chamber to the posterior segment and lead to blood–retinal barrier breakdown, resulting in an increased permeability of the foveal capillaries [2]. This accumulation of fluid leads to an increase in retinal thickness, which can result in pseudophakic cystoid macular edema (PCME), which is often associated with prolonged recovery time and impaired visual performance [3]. It is known that the retinal thickness increases following phacoemulsification, but it is still unclear which factors impact this the most [4,5,6,7,8,9]. The aim of this study is to investigate retinal thickness changes up to 12 weeks following surgery, and to identify their correlation with surgical and functional parameters. Thanks to optical coherence tomography (OCT), it is possible to non-invasively obtain high-resolution cross-sections of the retina and track changes occurring in its different layers. We set these changes into relation with the cumulative dissipated energy (CDE), total ultrasound time (UT), amount of fluids used, corrected distance visual acuity (CDVA), and intraocular pressure (IOP).

## 2. Materials and Methods

### 2.1. Patients

Forty-six eyes from forty-six patients scheduled to undergo routine cataract surgery at our center were enrolled in this prospective study. Adult patients with senile, traumatic, or iatrogenic cataracts were included. No patient had a spherical equivalent refraction greater than 8 dpt, a history of previous ocular surgery, an ocular condition (glaucoma, uveitis, retinal disorder, central corneal opacites, etc.) or systemic disorders affecting vision. The initial study population consisted of 47 patients, but one patient developed a pseudophakic cystoid macular oedema 4 weeks after surgery and was excluded. Only one eye was evaluated in this study. If both eyes required surgery, the one with the best OCT quality was selected. All patients were examined before surgery (T0), as well as 1 week (T1), 4 weeks (T2), and 12 weeks (T3) post-operatively. Each follow-up included Snellen CDVA, slit-lamp microscopy, Goldman applanation tonometry and a comprehensive fundus examination. This study was approved by the local ethics committee and conducted in accordance with the Declaration of Helsinki upon obtaining written informed consent from each participant.

### 2.2. Retinal Thickness and Lens Density Measurement

All retina cross-sections were obtained using the SPECTRALIS (Heidelberg Engineering GmbH, Heidelberg, Germany), a spectral-domain optical coherence tomography (SD-OCT) device, which, with a wavelength of 880 nm, has an axial resolution of 7 μm. Beforehand, full mydriasis was achieved in all patients with phenylephrine 5% and tropicamide eye drops to prevent shadowing due to the iris, and patients were instructed to stabilize their gaze on the fixation point and to keep the eye open. A square pattern centered on the macula consisting of 25 parallel B-scans of 6 mm length was used to obtain high-resolution cross-sections of the retina. To increase the image quality, a minimum of 40 B-Scans were averaged for each cross-section using the automatic real time mean (ART) algorithm built into the SPECTRALIS. In doing so, the signal-to-noise ratio of the image is continuously increased by the square root of the number of B-Scans, leading to improved contrast and reduced speckle pattern, enabling a clear visualization of the retina, even with dense cataracts [10]. Images with a quality index <30 were discarded, and the scan was repeated. The simultaneous registration of OCT and confocal scanning laser ophthalmoscopy (cSLO) images by the SPECTRALIS allows follow-up examinations to be at the exact same position, enabling a precise comparison of retinal morphology over an extended period of time [10]. All OCT scans were performed by a single experienced operator.

**Figure 1:**
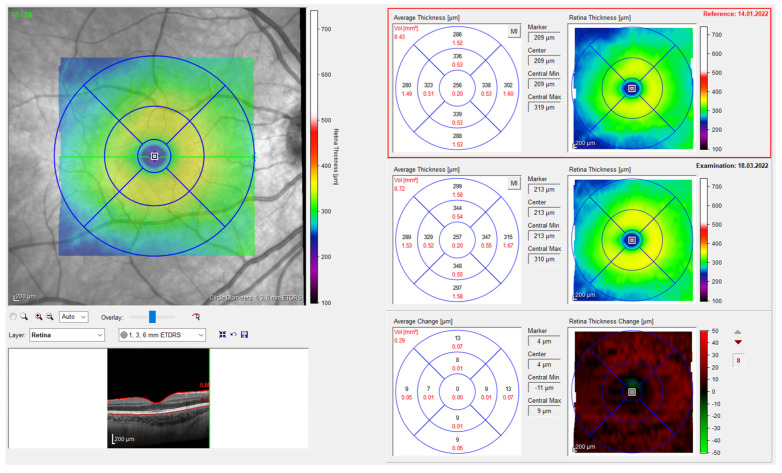
Measurement of the retinal thickness with the early treatment diabetic retinopathy study grid using spectral-domain optical coherence tomography (SD-OCT). The baseline color-coded measurement, showing the thickness of the retina, is shown in the top right field, followed by the newest measurement in the field below. The field in the bottom right displays the difference between both measurements.

The inbuild SPECTRALIS software is able to automatically detect the different retinal layers, enabling a precise segmentation of the retinal nerve fiber layer, the ganglion cell layer, the inner plexiform layer, the inner nuclear layer, the outer plexiform layer, the outer nuclear layer, the photoreceptor layer, and the retinal pigment epithelium [10]. In this study, we analyzed the entire retinal thickness, i.e., the distance between the internal limiting membrane and Bruch’s membrane. We used the early treatment diabetic retinopathy study (ETDRS) grid, which divides the retina into three rings: a central foveal ring with 1 mm diameter (CMT), an inner macular ring with 3 mm diameter (IMT), and an outer macular ring with 6 mm diameter (OMT) (Figure 1). Each ring is again divided into 4 quadrants (superior, inferior, nasal, and temporal), which we averaged to obtain the ring thickness. In addition to the individual thickness of each ring, we also recorded the retinal volume, i.e., the volume of the central disc with a 6 mm diameter. The automatic grid placement as well as the retinal segmentations were verified and if necessary corrected by an expert in the field.

The objective assessment of the crystalline lens density was achieved through anterior segment optical coherence tomography. The ANTERION (Heidelberg Engineering GmbH, Heidelberg, Germany) is a swept source OCT (SS-OCT) device that uses a laser with a wavelength of 1310 nm to produce cross-sections of the anterior segment with an axial resolution of <10 μm and a lateral resolution of 30 μm. The image quality is further improved by means of reduced speckle pattern by averaging up to 8 B-Scans thanks to the implementation of eye-tracking. All eyes were scanned following the retinal OCT examination by the same experienced operator. Patients were instructed to stabilize their gaze on the fixation point and to keep the eye open. A radial pattern of 15 B-Scans, each 14 mm long, was used to account for local variations in the lens anatomy. Scans that did not meet the inbuilt quality criteria were excluded, and the measurement was repeated. All images were then exported onto a personal computer for further analysis using a custom MATLAB (Version R2021b, MathWorks, Natick, MA, USA) script. Images were binarized using a general threshold following contrast adjustment. Noise removal and a morphological closing operation was applied to remove artifacts with the potential to affect the automatic lens segmentation. The lens boundaries were detected using the Canny edge detection algorithm and stray points were corrected using a polynomial of 4th order fitted to the lens border. The border of the lens nucleus was also segmented using this method. The density was then calculated by averaging the pixel density value of the segmented area and averaging the results of all 15 B-scans. To account for the background noise, the script subtracted the value of the anterior chamber density. This method has been validated in a previous study [11].

### 2.3. Surgical Technique

All patients underwent uneventful cataract surgery performed by two experienced surgeons using the divide-and-conquer technique. The Centurion Vision System (Alcon, Geneva, Switzerland) was used for phacoemulsification. This system automatically displays the CDE, UT, and amount of fluid used during the procedure. CDE is the total energy dissipated at the wound site during phacoemulsification and represents the energy needed to destroy the lens. Traditionally, the CDE is defined as CDE (%s) = Average phacoemulsification power (%) × Phacoemulsification time (s) [12], with time being the duration the foot pedal is activated. The LenSx femtosecond laser (Alcon, Geneva, Switzerland) was used on fourteen patients to perform capsulotomy and lens prefragmentation. No operative complication occurred, and no patient required pupillary manipulation. All patients received a post-operative 4-week-long treatment of neomycin and dexamethasone eye drops, which is the standard at our center. The one patient who developed a PCME was treated with Nepafenac, a nonsteroidal inflammatory drug, and made a complete recovery within 4 weeks.

### 2.4. Statistical Analysis

All results were analyzed using SPSS (Version 29, IBM, Chicago, IL, USA). Values are reported as means ± standard deviation. Pearson’s (r) correlation coefficient was used, as data normality was confirmed using the Shapiro–Wilk test. Additionally, Student’s paired-sample *t*-test was used to determine a significant difference in retinal thickness between the follow-ups. A *p*-value of 0.05 was considered significant. A pre-emptive sample size calculation showed that, assuming a true correlation between the macular thickness change and the surgical metric of 0.47, a sample size of 43 would have a power of 90% to detect a Pearson’s correlation coefficient different from 0, using a two-sided test at a significance level of 0.05.

## 3. Results

**Figure 2:**
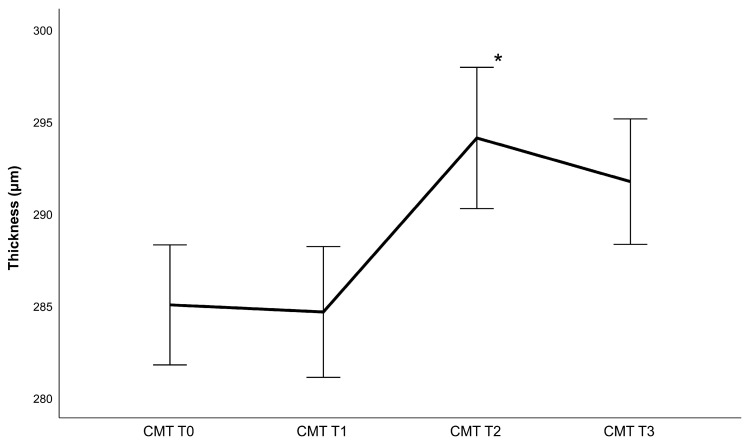
Central macular thickness (CMT) changes following phacoemulsification after one week (T1), one month (T2) and 3 months (T3) (mean ± standard error, * represents a statistically significant difference to the previous measurement with *p* < 0.001).

**Figure 3:**
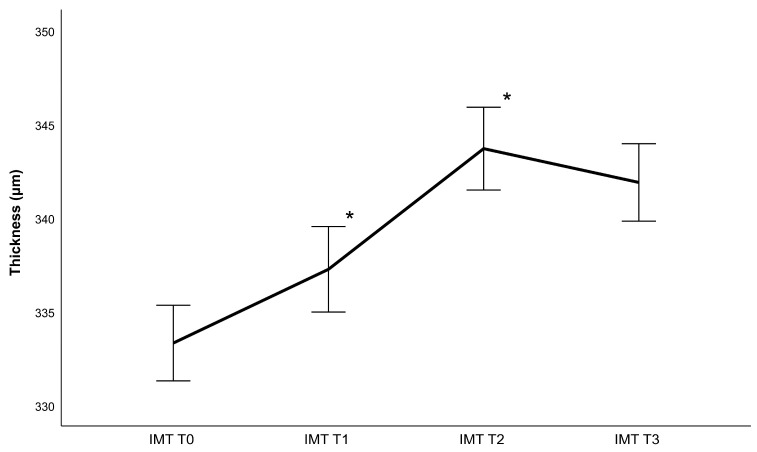
Inner macular thickness (IMT) changes following phacoemulsification after one week (T1), one month (T2) and 3 months (T3) (mean ± standard error, * represents a statistically significant difference to the previous measurement with *p* < 0.001).

**Figure 4:**
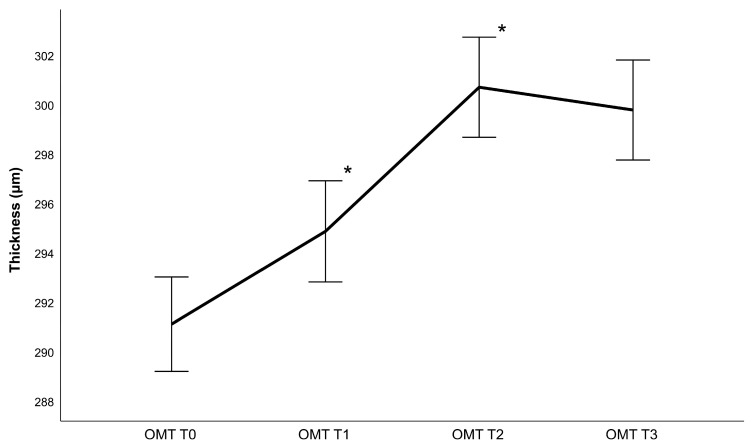
Outer macular thickness (OMT) changes following phacoemulsification after one week (T1), one month (T2) and 3 months (T3) (mean ± standard error, * represents a statistically significant difference to the previous measurement with *p* < 0.001).

**Figure 5:**
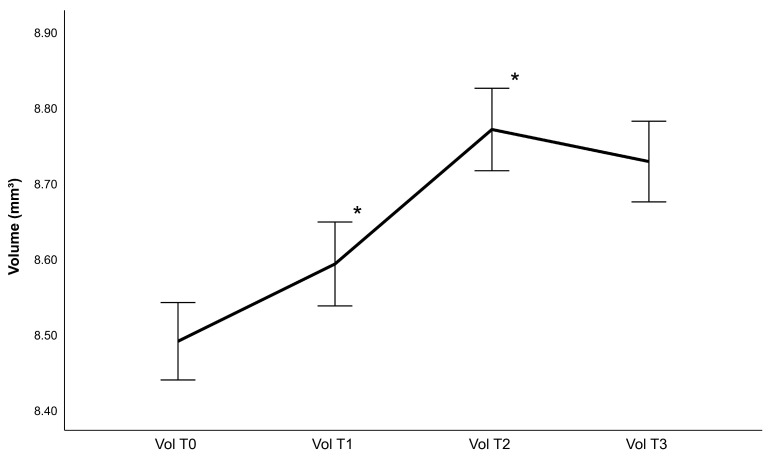
Retinal volume (Vol) changes following phacoemulsification after one week (T1), one month (T2) and 3 months (T3) (mean ± standard error, * represents a statistically significant difference to the previous measurement with *p* < 0.001).

Forty-six eyes of forty-six patients (22 women and 24 men) with an average age of 63 ± 10.25 years were examined in this study. As only one eye was selected, the dataset consisted of 26 right eyes and 20 left eyes. The CDVA and IOP measurements can be found in Table 1, while Table 2 contains lens density measurements and phacoemulsification parameters. Generally, the retina gained thickness following cataract surgery, achieving a maximum value 4 weeks post-operatively (Figure 2, Figure 3, Figure 4 and Figure 5). Thickness changes were statistically significant between all follow-ups, with the exception of T2 to T3, as well as T0 to T1 for the central macular thickness. There was no statistically significant difference between patients operated with and patients operated without the femtosecond laser.

Regarding phacoemulsification parameters, statistically significant correlations were found between the CDE and IMT, OMT and retinal volume change between T0 and T3 (r_IMT_ = 0.356 with *p* = 0.018, r_OMT_ = 0.298 with *p* = 0.049, r_volume_ = 0.357 with *p* = 0.017) as well as between T0 and T1 (r_IMT_ = 0.326 with *p* = 0.035, r_OMT_ = 0.351 with *p* = 0.023, r_volume_ = 0.333 with *p* = 0.031). Additionally, significant correlations were found between the ultrasound time and IMT, OMT, and retinal volume change between T0 and T3 (r_IMT_ = 0.369 with *p* = 0.032, r_OMT_ = 0.293 with 0.044 and r_volume_ = 0.409 with *p* = 0.016) as well as between T0 and T1 (r_IMT_ = 0.417 with *p* = 0.017 and r_OMT_ = 0.543 and r_volume_ = 0.516 with *p* < 0.01). There was no statistically significant correlation to retinal thickness changes between T1 and T2 as well as T2 and T3. There were also no significant correlations between retinal changes and the amount of fluid used, regardless of the area analyzed. Additionally, changes in the central macular thickness did not correlate significantly with any surgical parameters.

Regarding the functional metrics, there were significant correlations between nuclear density measurements and changes in OMT between T0 and T3 (r = 0.310, *p* = 0.036) as well as T0 and T1 (r = 0.376, *p* = 0.012) and volume changes between T0 and T1 (r = 0.331, *p* = 0.028). No statistically significant correlations were found regarding lenticular density, changes in CDVA or changes in tonometry.

## 4. Discussion

**Figure 6:**
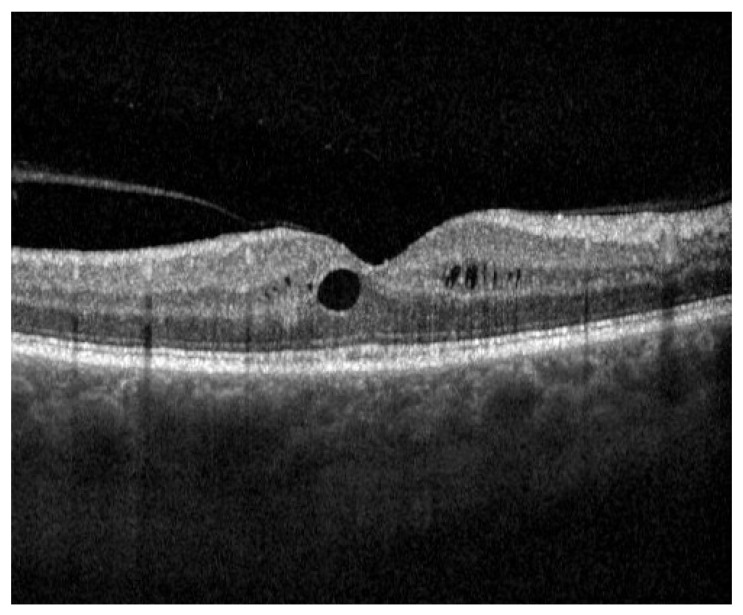
Pseudophakic cystoid macula edema (PCME), identified by the dark areas representing intraretinal fluid, occurring 4 weeks after cataract surgery imaged with spectral-domain optical coherence tomography (SD-OCT). Vitreous body detachment can accessorily be seen on the left side of the image.

The results of the current study demonstrate a significant increase in retinal thickness following phacoemulsification and intraocular lens implantation. The literature describes the incidence of PCME to range from 0.1% to 2.35% in the general population, occurring around 4 to 6 weeks post-operatively [13]. This is in line with our findings, as one patient (2.12% of the study population) developed subclinical macular oedema 4 weeks after surgery (Figure 6). Complete remission was achieved 4 weeks later under 0.1% nepafenac eye drop therapy. Interestingly, peak retinal thickness in this study occurred 4 weeks after surgery, before dropping to a lower, albeit still significantly higher than before surgery, thickness in the following months. This falls in line with the results of previous studies, and strengthens the intuitive idea that inflammation is highest in the first weeks following surgery [4,5,6,7,8,9]. While the clinical significance of this increase in healthy individuals seems to be negligible, it can pose a risk for certain patients suffering from diabetes or retinal disorders, such as age-related macular degeneration [3]. Although considered a risk factor for PCME, Brito et al. showed that there were no statistically significant differences in macular thickness between eyes with diabetic retinopathy and controls after cataract surgery [14]. Ikegami et al. found similar results with patients suffering from diabetes [6]. However, when examining the aqueous flare value, an indicator for the degree of inflammation, they noted a significant increase in the diabetes cohort compared to the control group [6]. This suggests that an increase in inflammation alone is not sufficient to trigger the occurrence of PCME and that, while playing a part, it is not the only reason for retinal swelling. Still, anti-inflammatory drugs play a vital role in the prevention of PCME [3]. Interestingly, the thickness of the macula itself (inner 1 mm circle) did not change significantly in the first week following surgery. This might be due to the fact that the foveal avascular zone (FAZ), a region of the retina devoid of blood vessels, is located in the macula. Therefore, it is less impacted by the blood–retina barrier breakdown. However, the macula thickness did increase significantly in the following weeks, indicating that other factors apart from inflammatory mediators play a role in retinal thickness evolution.

As the lens opacity increases, the visual acuity as well as the total amount of light that reaches the retina decreases, explaining patients’ reported light discomfort in the first days after surgery. This increase in light leads to an increase in retinal metabolism, which necessitates more oxygen, leading to a rise in blood flow and blood vessel caliber. Indeed, Norman et al. found a link between retinal metabolism and retinal thickness in rats [15]. Furthermore, Lou et al. discovered that light deprivation has an effect on retinal thickness, while having no effect on choroidal thickness [16]. To what extent this change in light transmission following intraocular lens implantation impacts the retinal metabolism needs to be explored in a future study. Meanwhile, no link between retinal thickness and CDVA increase has been found in the current work. However, we did find a correlation between nuclear density and retinal thickness increase. Whether this is due to the increase in light transmission after surgery, or the CDE used during the operation, is questionable, as it is unclear how strongly our nuclear density measurement reflects the light transmission of the lens.

During cataract surgery, the ultrasound energy used for phacoemulsification leads to the creation of hydroxyradicals, which in turn leads to corneal endothelial damage and acute inflammation processes [17]. We found that both the CDE and the UT correlated with retinal thickness increase, which is in line with the literature [18]. The highest correlation was found with the thickness increase directly after surgery, which is to be expected, as this is when inflammation due to the ultrasound energy is the highest. It is known that the use of the femtosecond laser can effectively reduce the CDE [19], and in turn, reduce the retinal thickness increase. Therefore, femtosecond laser assisted cataract surgery (FLACS) might be beneficial to patients who have an increased risk of PCME, as the inflammatory strain following surgery is reduced. Meanwhile, the amount of fluid used during surgery did not show any link to macular volume increase. This is possibly due to the fact that modern phacoemulsification machines better control the intraocular pressure during surgery, making it possible to operate at nearly physiological pressure levels. Therefore, the strain on the ocular structures is again kept minimal, keeping the risk of PCME low.

A limitation of this work is the relatively short follow-up time. While we did show a thickness decrease at the end of the 3-month period, it is unclear how the retinal thickness evolves after this point. In a study by Yilmaz et al., it was shown that macular thickness returns to preoperative values after 12 months, suggesting that changes are only transient due to inflammation [20]. Choroidal thickness, on the other hand, did not return to its baseline value, which might play a role in certain ailments such as late-onset age-related macular degeneration.

## 5. Conclusions

The current study demonstrated that retinal thickness significantly increases following uneventful cataract surgery. Furthermore, the significant correlation between macular thickness increase and cumulative dissipated energy as well as ultrasound time was documented, indicating that surgical technic and phacoemulsification parameters influence blood-retinal barrier integrity and contribute to PCME formation. No correlation was found between thickness increase and CDVA or IOP. Modern cataract surgery strides towards ever less-straining techniques, minimizing post-operative inflammation levels, and reducing the risk of PCME. How the retinal metabolism evolves following surgery, and how it influences macular integrity and function, needs to be explored further.

## Figures and Tables

**Table 1:** Overview of corrected distance visual acuity and intraocular pressure values.

Corrected distance visual acuity mean ± 1 SD	Pre-OP	0.62 ± 0.21
	1 Week	0.85 ± 0.19
	4 Weeks	0.92 ± 0.17
	12 Weeks	0.97 ± 0.17
Intraocular pressure mean ± 1 SD (mmHg)	Pre-OP	16.08 ± 3.25
	1 Week	15.40 ± 4.52
	4 Weeks	14.30 ± 2.44
	12 Weeks	12.96 ± 2.95

Pre-OP = Pre-operative value; SD = Standard deviation.

**Table 2:** Lenticular and surgical data.

	Mean ± 1 SD
Lens Density (Pixel Intensity Value)	43.92 ± 6.92
Nuclear Density (Pixel Intensity Value)	36.72 ± 13.67
Cumulative Dissipated Energy (%s)	2.89 ± 1.80
Ultrasound Time (s)	22.24 ± 10.78
Fluids Used (mL)	41.81 ± 12.92

SD = Standard deviation.

## Data Availability

All available data generated or analyzed during this study are included in this published article.
